# Loneliness in the Republic of Srpska: advocating for social prescribing

**DOI:** 10.1093/eurpub/ckae148

**Published:** 2024-09-29

**Authors:** Sonja Stančić, Strahinja Dimitrijević, Dragana Vidović, Arijana Radić

**Affiliations:** Department of Psychology, Faculty of Philosophy, University of Banja Luka, Banja Luka, Bosnia and Herzegovina; Department of Psychology, Faculty of Philosophy, University of Banja Luka, Banja Luka, Bosnia and Herzegovina; Department of Government, University of Essex, Colchester, United Kingdom; Department of Psychology, Faculty of Philosophy, University of Banja Luka, Banja Luka, Bosnia and Herzegovina

## Abstract

This study explores the potential implementation of social prescribing in the Republic of Srpska, Bosnia and Herzegovina, where the approach is non-existent, and supporting structures are underdeveloped despite a recognized need for intervention. As social prescribing gains global recognition for improving health, the study investigates its feasibility in an uncharted area. The research assesses the necessity for social prescribing by examining loneliness rates and healthcare utilization in the Republic of Srpska, a region seldom studied in public health literature. Data from 1231 individuals aged 16–86 were collected in May 2021, marking the first initiative to gather information on loneliness and healthcare usage in the country. Loneliness rates in the Republic of Srpska were comparable to the UK. Using a negative binomial model, the study establishes significant links between loneliness, chronic health conditions, age, and healthcare service utilization. Loneliness, chronic health conditions, and age predict the use of general practitioner services. In the 44–54 and 65+ age groups, loneliness predicts accident and emergency service use. Specialist healthcare services are positively predicted by loneliness, having one chronic health condition, and being above 44 years of age. Notably, a COVID-19 diagnosis negatively predicts the use of all healthcare services. Gender and place of residence do not significantly impact healthcare service utilization. The study concludes that observed loneliness rates and correlated healthcare usage patterns in the Republic of Srpska indicate a need for social prescribing. The paper discusses the feasibility of implementing social prescribing in this particular case.

Key points

- Loneliness, chronic conditions, and age impact healthcare utilization, emphasizing social determinants’ role.
- There is a clear need for social prescribing in the Republic of Srpska.
- Successful social prescribing hinges on understanding and integrating health structures effectively with other public services.
- Empowering communities is vital for tackling health inequalities through social prescribing.

## Introduction

The social prescribing model is becoming a global response to public health challenges [1]. The rise in the popularity was driven by the reports that a large number of medical appointments are due to non-medical issues such as loneliness and poor well-being [2–4]. The model is perceived as a means to enhance health, well-being, and community ties [5–8] by allowing health and social care professionals to recognize non-medical, health-related social needs and refer individuals to various non-clinical services within the community [5]. The model integrates health and social care, local authority, voluntary, community and social enterprise sector organizations (VCSE), and other public sectors.

While a number of reports show that social prescribing has a positive impact on individual health outcomes and usage of health services, some suggest that the effectiveness of this model requires further review [2, 7, 9–14]. Social prescribing has been primarily implemented in the UK, where it originated [15], and is becoming more widely implemented across the globe [1, 5, 16]. Thus, more work is needed to examine if and to what extent social prescribing is comparable and feasible across various contexts.

In the current study, we explore an argument for the implementation of a social prescribing model in the Republic of Srpska, where this approach is relatively unexplored. We examine the prevalence of loneliness and healthcare usage using newly collected data from the Republic of Srpska, a rarely studied case in the public health literature.

Implementing social prescribing involves collaboration among the VCSE sector, health and social care services, local authorities, and social prescribing professionals. Social prescribing professionals are trained to support individuals to better understand their needs and link them with the resources available in their communities to improve and manage their health [3, 7]. In order for referrals to be made in a timely and effective manner, cross-sectoral partnership and communication are essential. Furthermore, the structures to support the development of the workforce, impact assessment [16], and legislative frameworks are needed for successful delivery.

In the current study, we address the following questions in regard to the case of the Republic of Srpska: (i) Is there a need for social prescribing?; (ii) Are there structures to implement it?; and (iii) Is it feasible to implement social prescribing in the Republic of Srpska?

### Social prescribing and public health

Social prescribing is an asset-based, holistic, health coaching scheme designed to address a complex set of physical, mental, and social care needs, offering community solutions alongside medical answers to health issues [2, 5]. Initially, it was seen as a way for general practitioners (GPs) in the UK to reduce pressure on GP services by referring individuals to a range of non-medical services to address loneliness, social isolation, and poor well-being. Other professionals in public health (e.g. social care) also recognize the value of social prescribing and utilize this model [13]. Yet, the extent of the networks involved in delivering social prescribing is understudied in the current literature.

In England, where social prescribing is prominent, the integration of health and social care systems with the VCSE sector, to offer personalized care is currently being guided by the Health and Care Act 2022 [17]. Personalized care is based on working to understand ‘what matters’ to people, identifying their individual strengths and needs, as one-size-fits-all health and care system simply cannot meet the increasing complexity of people’s needs and expectations. National Health Services (NHS) England and NHS Improvement have published guidance for including the VCSE sector into the partnership of health and care organizations, known as integrated care systems (ICSs) [17]. This represents a significant step in establishing partnerships based on equity in decision-making and governance and developing a whole-system approach to address public health needs.

In the Republic of Srpska, healthcare legislation (Official Gazette of RS, No. 57/2022) outlines the rights and responsibilities of the healthcare sector and citizens. The law on social care (Official Gazette of the RS, No. 36/2022) defines social care as a preventative and protective activity for those in need. Various strategies, such as the 2018–29 Elderly Persons Improvement Strategy [18], the Social Cohesion and Inclusion Strategy [19], and the 2023–27 Youth Policy, aim to enhance the well-being of citizens, especially those in vulnerable communities. However, despite the existing framework, there is a lack of coordinated action to address specific issues or target groups. Exploring integrated models, such as the UK’s social prescribing, could be beneficial for enhancing care in the Republic of Srpska.

Social prescribing is crucial to a sustainable health system as it recognizes social, economic, and environmental factors as health determinants. Social prescribing professionals guide individuals to engage with diverse community services, including arts, sports, education, training, social events, to simply assisting local elderly residents with gardening. Social prescribing goes beyond addressing individual needs as it seeks to build a personal and social capacity to adaptively and proactively respond to challenges facing individuals and communities across the globe. By participating in these and similar social activities, individuals are likely to increase their social connections, become more integrated in their communities, and become more aware of the resources that they could utilize to improve their health outcomes [20]. A social prescribing program could support an individual to better manage their long-term health condition while simultaneously providing support regarding one’s employment or housing needs, thus enhancing one’s social and economic prospects, one’s quality of life, and as a result, a person’s ability to actively contribute to life in their community. Society as a whole is likely to benefit when circumstances improve at an individual level, with potentially positive changes in economic, social, and political outlooks of a community as a whole.

Community in which individuals live, work, and grow, is an important social determinant of health. Community-based models such as social prescribing can help individuals better understand existing resources and issues facing their community, all of which could increase access to more and better quality of care as well as one’s knowledge and skills on how to access it. Social prescribing enables a more coordinated and proactive care, where individuals are empowered to consider their health needs and seek solutions within an ICS with an aim to optimize resources and receive more adequate care. The model plays a vital role in addressing wider health determinants, reaching individuals from diverse backgrounds and empowering them to leverage community resources and overcome barriers to accessing services.

### Social prescribing and loneliness

Loneliness is a subjective feeling of a lack of companionship which can be experienced even when surrounded by others, where there is a mismatch between the quality and quantity of relationships one has versus those that one wishes to have [21, 22].

This phenomenon is acknowledged as a public health crisis negatively impacting individual health and putting additional pressure on already overtaxed public services across the globe [21, 23–25]. The focus on social health needs and the wider social determinants of health have promoted a broader debate about the reforms in the health and social care sector. Social prescribing is viewed as an essential approach to addressing public health crises such as loneliness and reforming the health and social care sector.

It has been found to negatively impact numerous physical and mental health outcomes, including cardiovascular health, cognitive functioning, substance abuse, poor decision-making as well as being linked to early mortality [21, 23, 24, 26]. Social connections and support of others matter in many aspects of life, from engaging in healthy behaviors such as group exercise to a feeling of belonging to one’s community, all of which help improve health. Lonely individuals are more likely to exhibit poor immune functioning, changes in stress hormones, and cardiovascular system health [21]. Incidents of coronary heart disease and stroke are higher in individuals with poor social relationships as well as the cardiovascular disease prognosis [27]. Numerous research studies have shown a link between physical health and a lack of meaningful social connections, and thus, social health issues such as loneliness should be considered by medical health professionals during physical health examinations. The European Commission 2021 report indicates that loneliness is more prevalent in Eastern and Southern Europe in comparison to Western and Northern Europe [28]. This further justifies the need to focus on the region of Southern Europe, the Republic of Srpska, an area which is greatly understudied in the current literature.

Various types of interventions are used to address loneliness [29]. While majority of the studies report a positive impact of various models of intervention on loneliness, the critique in the current literature is that the results are not easily generalizable due to a variability of methods used to assess the impact of interventions on loneliness [29, 30]. Despite some of its shortcomings, the social prescribing model is becoming a globally accepted standard to address loneliness and improve public health [1, 5, 16].

### Case study: Republic of Srpska, public health, and social prescribing

The World Bank 2019 report indicates that the population health in Bosnia and Herzegovina is likely to worsen if service design and delivery, facility management, and financing issues are not addressed [31]. The complex geographical, political, and administrative divisions, set following the 1995 Dayton Peace Agreement, have resulted in three separate territorial units and health and social care systems, with significant impacts on governance and public policy implementation [32]. The complexities that come with implementing research in each region and a lack of coordination across various health and research sectors are further contributing to the worsening public health.

Health and social care systems in the Republic of Srpska, one of Bosnia and Herzegovina’s territorial units, lack an integrated legislative framework to support practice in both sectors. The needs of the population and changing life circumstances necessitate more adaptable and integrated responses to public health needs; however, there is minimal overlap and joint activity between the health and social care sectors. The Ministry of Health and Social Welfare of the Republic of Srpska has recognized this issue and appointed a working group which drafted the Social Inclusion Strategy 2023–29 [18], bringing together civil society and public health organizations to work on creating effective and responsive health and social systems better prepared to address emerging public health needs.

**Figure 1.**
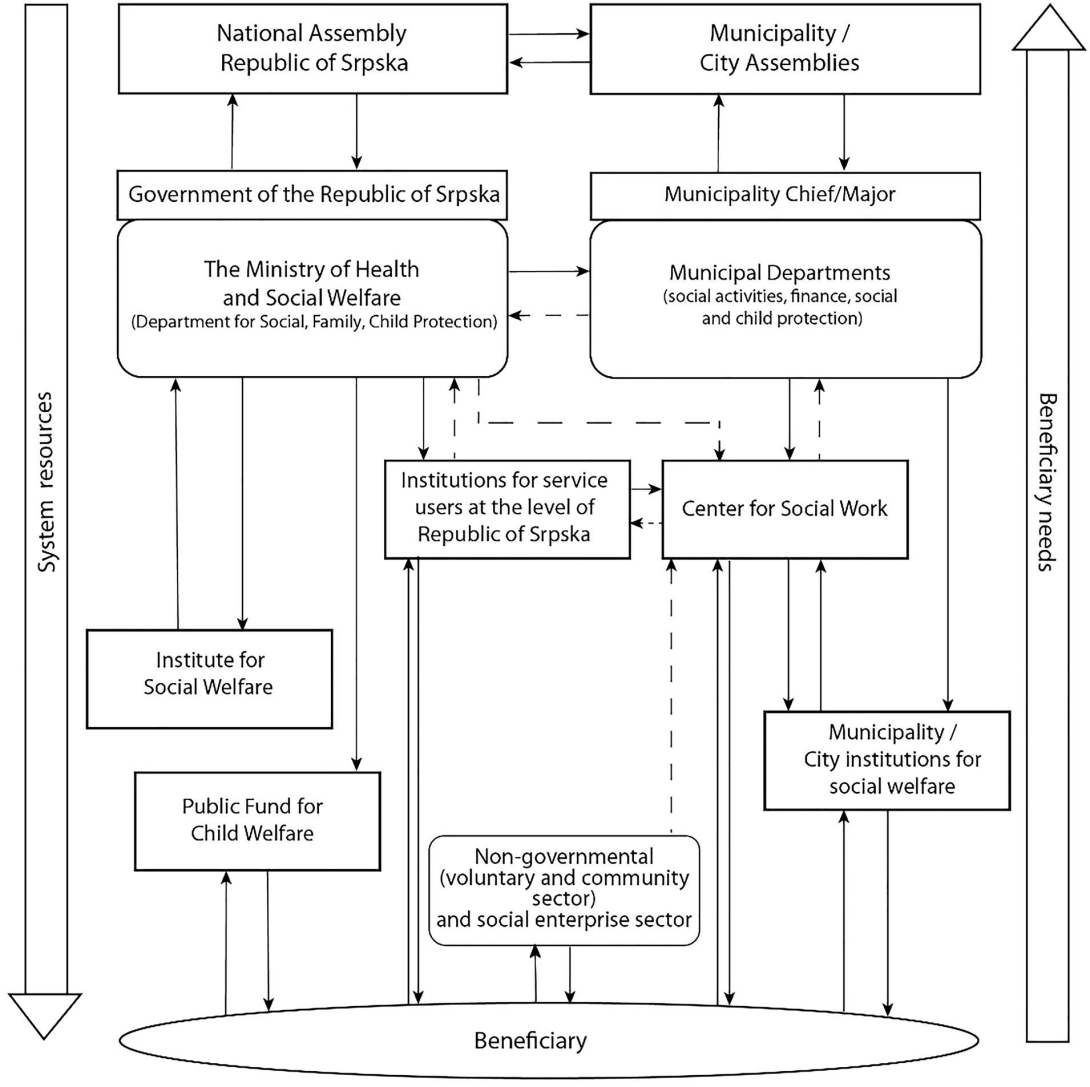
Social care system governance in the Republic of Srpska. The image represents the institutional framework of the social, child, and family protection system in the Republic of Srpska. It illustrates both horizontal and vertical communication between all actors in the social protection system: from the executive and legislative authorities at the entity and local levels, through public institutions, non-governmental organizations, to the users. This system is significant as it aims to address issues such as loneliness within its framework. The chart was sourced from https://shorturl.at/w2sro.

Civil sector organizations (similar to the VCSE in the UK) are often the first point of contact when addressing social needs, as Fig. 1 shows. However, limited financial resources have led to a decline in support, putting pressure on health and social care services and endangering the vulnerable. Greater cooperation and integration across sectors are needed to address rising health and social care needs. The social prescribing model could exemplify such cooperation and integration in the Republic of Srpska.

The ‘Banjaluka with a Human Face’ initiative [33], showcases an example of cross-sectoral collaboration. It serves as a unique example in the country, resembling the social prescribing model in the UK. The initiative unites professionals from over 20 citizens’ associations, educational institutions, governmental and non-governmental organizations, health and social care sectors, and citizens. Together, they address diverse health needs, promoting physical, mental, and social well-being. The network aims to optimize resource management, foster information sharing, and co-produce activities aligned with citizens’ needs. All activities organized in collaboration with network members are free, prioritizing the enhancement of individual health and well-being.

The ‘The ‘Banjaluka with a Human Face’ initiative [33] addresses diverse needs, including improving the well-being of children and youth through organized activities, enhancing the quality of life for individuals aged 65 and above with cultural and social engagements, and supporting individuals with disabilities. Despite challenges like COVID-19, which is a contagious disease caused by the coronavirus SARS-CoV-2, the initiative sustained its activities online or in-person. This study explores the need for social prescribing on the basis of levels of loneliness and its impact on healthcare utilization. Cardiovascular diseases account for the largest percentage of chronic health problems in the Republic of Srpska (19.81%) [34]. Numerous socio-economic and political factors in the country can be linked to the prevalence of poor cardiovascular health. While finding resources to address these complex societal-level factors might be challenging, targeting loneliness as one of the issues underlying health is likely to be more attainable. Engaging in healthy behaviors, exercising, being outdoors, socializing and connecting with others are actions that individuals have more control over and can participate in given adequate community-level support [8, 27]. Given the links between loneliness, cardiovascular health, and utilization of healthcare services, addressing loneliness as a public health issue in the Republic of Srpska is of utmost importance.

## Methods

The study, conducted in May 2021, included 1231 respondents (aged 16–86; average age = 42) from the Republic of Srpska, of whom 55.6% were women. It comprised respondents from rural areas (18.1%), suburban areas (21.6%), and urban areas (60.3%). The participants were recruited by advertising via local public boards and the University of Banja Luka. The ‘Banjaluka with a Human Face’ initiative [33] survey advertisements. An online platform called 1KA (https://www.1ka.si) was used to enable survey data collection and recording. To the best of our knowledge, this is the first of its kind effort to collect data on loneliness, healthcare usage, and related issues in the region [35].

A negative binomial regression model [36] for modeling count variables was used to examine the links between loneliness, chronic health conditions, and healthcare usage rates. In this model, loneliness was treated as the primary variable of interest, while age, COVID-19 diagnosis, gender, and place of residence were included as control variables. This means that the model accounts for these factors to isolate the specific effect of loneliness on healthcare usage.

### Dependent variables

We examine three count-dependent variables to assess healthcare services usage. Respondents were asked to recall their level of healthcare usage (GP, accident and emergency [A&E], and healthcare specialist visits), ranging from 0 to 16+ in the last month. Healthcare specialist roles are most similar to the consultant role in the NHS system, with a specific specialty (e.g. vascular, endocrine, gastrointestinal, musculoskeletal).

### Independent variables

Loneliness was assessed using a scale that asked respondents, ‘How often do you feel lonely?’ The five answer options were: Never, Hardly ever, Occasionally, Some of the time, and Often/always. To facilitate analysis, the scale was reversed, coding Never as 1, Hardly ever as 2, Occasionally as 3, Some of the time as 4, and Often/always as 5. The participants were asked to list all chronic illnesses they had been diagnosed with. The responses were categorized into four groups: 0–1–2–3 or more. This approach aimed to capture chronic diagnoses that could affect both current health status and the use of healthcare services. Chronic health conditions included those linked to loneliness and/or increased usage of healthcare services: asthma, arthritis, diabetes, epilepsy, overweight, heart and coronary issues, blood pressure, cancer, lung conditions, musculoskeletal issues, migraine, stroke, anxiety, depression, chronic stress, and personality-related disorders.

### Control variables

We controlled for potential confounding factors such as gender, age, place of residence, and COVID-19 diagnosis.

### Ethical issues

In our study, we prioritized ethical considerations to maintain research integrity. All participants gave informed consent, understanding the study’s purpose, risks, and their rights. Privacy protection measures were in place, including a recruitment process via public boards and university advertisements. Acknowledging potential bias, especially for those without internet access, we reversed the loneliness scale for analysis, considering its psychological implications. Careful handling of sensitive health data and transparent communication about COVID-19 information underscore our commitment to ethical practices. Our study, approved by an ethical review board, upholds high standards, ensuring transparency and credibility in research involving human subjects.

## Results

Loneliness prevalence in this study is similar to the rates of loneliness in the UK and other Western countries. Among respondents in the current study, 4.7% frequently feel lonely, 6.6% experience loneliness some of the time, 32.8% occasionally feel lonely, 37.9% rarely feel lonely, and 18% report never feeling lonely. Examining loneliness across age groups reveals that individuals aged 65 and above report the highest levels, with 10.2% feeling often/always lonely. Following this group, the age categories of 45–54 (4.7%), 55–64 (4.5%), 25–34 (3.9%), and 16–24 (2.9%) experience varying degrees of loneliness, with individuals in the 35–44 age category reporting the lowest levels at 2.8%.

In terms of chronic conditions, 50.5% of individuals report not having any, 26.9% report having one chronic condition, 13.2% report having two, and 9.4% report having three or more chronic conditions.

For each unit increase in loneliness score, the likelihood of visiting a GP increases by an exponential coefficient of 1.12, a statistically significant difference (*P* < .05), holding all other variables constant (Table 1). In addition, chronic health conditions and age are positive and significant predictors of GP healthcare service utilization, while a COVID-19 diagnosis is a negative and significant predictor. Gender and place of residence do not significantly impact the number of GP visits.

**Table 1.** Negative binomial model: loneliness, chronic health conditions, and GP usage

					95% Exp(*B*) confidence interval			
Names	Effect	Estimate	SE	Exp(*B*)	Lower	Upper	*z*	*P*
(Intercept)	(Intercept)	−0.56	0.15	0.58	0.42	0.80	−3.44	<.001
Loneliness	Loneliness	0.11	0.05	1.11	0.99	1.24	2.04	.04
Gender (1—female)	2-1	−0.04	0.11	0.96	0.77	1.20	−0.32	.74
Age2 (25–34)^a^	2-1	0.56	0.19	1.74	1.18	2.57	2.81	.005
Age3 (35–44)^a^	3-1	0.48	0.21	1.62	1.05	2.49	2.22	.02
Age4 (44–54)^a^	4-1	0.99	0.19	2.69	1.84	3.97	5.09	<.001
Age5 (55–64)^a^	5-1	1.19	0.22	3.30	2.14	5.13	5.41	<.001
Age6 (65+)^a^	6-1	1.48	0.19	4.39	2.98	6.54	7.45	<.001
Residence2 (suburban)^b^	2-1	0.13	0.16	1.14	0.81	1.60	0.79	.42
Residence3 (urban)^b^	3-1	−0.05	0.14	0.95	0.71	1.26	−0.34	.73
COVID 19 (1—yes; 2—no)	1-2	0.57	0.12	1.76	1.38	2.25	4.72	<.001
ChronicHelthC.2 (one)^c^	1-0	0.54	0.13	1.71	1.30	2.23	3.93	<.001
ChronicHelthC.3 (two)^c^	2-0	0.69	0.17	1.99	1.41	2.80	4.03	<.001
ChronicHelthC.4 (three+)^c^	3-0	0.85	0.18	2.35	1.64	3.38	4.70	<.001

a Age 1 (16–24) comparison category.

b Residence 1 (rural) comparison category.

c Chronic Health Condition 1 (none) comparison category.

The results shown in Table 2 indicate that for each unit increase in loneliness score, the likelihood of using A&E services increases by an exponential coefficient of 1.26, a statistically significant difference (*P* < .01), holding all other variables constant (Table 2). As well as in the case of GP visits. Age was a significant and positive predictor of utilizing A&E healthcare services, while a COVID-19 diagnosis is a negative and significant predictor. Gender and place of residence do not significantly impact the number of A&E visits.

**Table 2.** Negative binomial model: loneliness, chronic health conditions, and A&E usage

					95% Exp(*B*) confidence interval			
Names	Effect	Estimate	SE	Exp(*B*)	Lower	Upper	*z*	*P*
(Intercept)	(Intercept)	−1.36	0.24	0.25	0.15	0.42	−5.65	<.001
Loneliness	Loneliness	0.23	0.08	1.26	1.05	1.51	2.83	.005
Gender (1—female)	2-1	0.24	0.16	1.27	0.91	1.78	1.45	.14
Age2 (25–34)^a^	2-1	−0.22	0.28	0.80	0.45	1.40	−0.78	.43
Age3 (35–44)^a^	3-1	0.06	0.30	1.07	0.58	1.97	0.22	.82
Age4 (44–54)^a^	4-1	0.80	0.26	2.24	1.31	3.85	3.00	.003
Age5 (55–64)^a^	5-1	0.59	0.32	1.81	0.94	3.52	1.80	.07
Age6 (65+)^a^	6-1	1.23	0.28	3.43	1.94	6.11	4.37	<.001
Residence2 (suburban)^b^	2-1	−0.21	0.25	0.80	0.47	1.34	−0.86	.38
Residence3 (urban)^b^	3-1	−0.39	0.21	0.67	0.43	1.03	−1.84	.06
COVID 19 (1—yes; 2—no)	1-2	0.60	0.18	1.83	1.25	2.71	3.24	.001
ChronicHelthC.2 (one)^c^	1-0	0.25	0.20	1.29	0.84	1.96	1.23	.21
ChronicHelthC.3 (two)^c^	2-0	0.38	0.26	1.46	0.85	2.54	1.44	.14
ChronicHelthC.4 (three+)^c^	3-0	0.22	0.29	1.25	0.70	2.27	0.77	.43

a Age 1 (16–24) comparison category.

b Residence 1 (rural) comparison category.

c Chronic Health Condition 1 (none) comparison category.

With each unit increase in the loneliness score, the likelihood of using specialist healthcare services increases by a factor of 1.15, which is a statistically significant difference (*P* < .05), while holding all other variables constant (Table 3). Having one chronic health condition, and being over 44 years of age are significant and positive predictors of using specialist healthcare services, while a COVID-19 diagnosis is a negative and significant predictor. Gender and place of residence do not significantly impact the number of specialist visits.

**Table 3.** Negative binomial model: loneliness, chronic health conditions, and usage of specialist healthcare services

					95% Exp(*B*) confidence interval			
Names	Effect	Estimate	SE	Exp(*B*)	Lower	Upper	*z*	*P*
(Intercept)	(Intercept)	−1.36	0.24	0.25	0.15	0.42	−5.65	<.001
Loneliness	Loneliness	0.23	0.08	1.26	1.05	1.51	2.83	.005
Gender (1—female)	2-1	0.24	0.16	1.27	0.91	1.78	1.45	.14
Age2 (25–34)^a^	2-1	−0.22	0.28	0.80	0.45	1.40	−0.78	.43
Age3 (35–44)^a^	3-1	0.06	0.30	1.07	0.58	1.97	0.22	.82
Age4 (44–54)^a^	4-1	0.80	0.26	2.24	1.31	3.85	3.00	.003
Age5 (55–64)^a^	5-1	0.59	0.32	1.81	0.94	3.52	1.80	.07
Age6 (65+)^a^	6-1	1.23	0.28	3.43	1.94	6.11	4.37	<.001
Residence2 (suburban)^b^	2-1	−0.21	0.25	0.80	0.47	1.34	−0.86	.38
Residence3 (urban)^b^	3-1	−0.39	0.21	0.67	0.43	1.03	−1.84	.06
COVID 19 (1—yes; 2—no)	1-2	0.60	0.18	1.83	1.25	2.71	3.24	.001
ChronicHelthC.2 (one)^c^	1-0	0.25	0.20	1.29	0.84	1.96	1.23	.21
ChronicHelthC.3 (two)^c^	2-0	0.38	0.26	1.46	0.85	2.54	1.44	.14
ChronicHelthC.4 (three+)^c^	3-0	0.22	0.29	1.25	0.70	2.27	0.77	.43

a Age 1 (16–24) comparison category.

b Residence 1 (rural) comparison category.

c Chronic Health Condition 1 (none) comparison category.

## Conclusions

Numerous research studies indicate that social health issues, notably loneliness, significantly impact health, correlating with increased healthcare utilization. This study affirms this correlation in the Republic of Srpska, an underexplored region in public health literature. The necessity for social prescribing is evident, suggesting that changes in practical and policy approaches could facilitate its implementation, enhancing public health and health and social care services. Social prescribing allows for detailed mapping of social health needs, particularly loneliness, an aspect often overlooked in public health reports. This effort represents the first data collection on loneliness prevalence in the region. Social prescribing establishes a framework for communities to develop and expand resources, addressing complex public health needs. It may spawn new professions, avenues for academic research, and training in public health. Introducing social prescribing in the Republic of Srpska promises to enhance public health, conserve resources, and foster developmental opportunities.

Conflict of interest: None declared.

## Data Availability

The data underlying this article will be shared on reasonable request to the corresponding author.
