# Correction to: Loneliness in the Republic of Srpska: advocating for social prescribing

**DOI:** 10.1093/eurpub/ckaf142

**Published:** 2025-09-01

This is a correction to: Sonja Stančić, Strahinja Dimitrijević, Dragana Vidović, Arijana Radić, Loneliness in the Republic of Srpska: advocating for social prescribing, *European Journal of Public Health*, Volume 34, Issue 6, December 2024, Pages 1073–1078, https://doi.org/10.1093/eurpub/ckae148

In the originally published version of the manuscript, Table 3 was mistakenly duplicated from Table 2. As a result, the values relating to specialist healthcare service use were not correctly presented in the table, although the text reflects the correct results. The correct exponential coefficient for loneliness in the model is 1.15. Table 3 has been emended within the article.

